# An illustrated approach to Soft Textual Cartography

**DOI:** 10.1007/s41109-018-0087-y

**Published:** 2018-08-13

**Authors:** Raphaël Ceré, Mattia Egloff

**Affiliations:** 1 Department of Geography and Sustainability, University of Lausanne, Lausanne, Switzerland; 2 Department of Language and Information Sciences, University of Lausanne, Lausanne, Switzerland

**Keywords:** Textual cartography, Complex network, Topic modelling, Thematic exploration, Soft clustering, Text mining, GIS, Membership association, Wikipedia

## Abstract

We propose and illustrate an approach of Soft Textual Cartography consisting in the clustering of regions by taking into account both their spatial relationships and their textual description within a corpus. We reduce large geo-referenced textual content into topics and merge them with their spatial configuration to reveal spatial patterns. The strategy consists in constructing a complex weighted network, reflecting the geographical layout, and whose nodes are further characterised by their thematic dissimilarity, extracted form topic modelling. A soft k-means procedure, taking into account both aspects through expectation maximisation on Gaussian mixture models and label propagation, converges towards a soft membership, to be further compared with expert knowledge on regions. Application on the Wikipedia pages of Swiss municipalities demonstrate the potential of the approach, revealing textual autocorrelation and associations with official classifications. The synergy of the spatial and textual aspects appears promising in topic interpretation and geographical information retrieval, and able to incorporate expert knowledge through the choice of the initial membership.

## Introduction

Regional data analysis generally involves numerical or categorical information attached to the regions, such as level intensities or densities provided from census data (e.g. population, socio-economical properties). Another rich information source that should be considered in regional data analysis is “common textual knowledge”. Yet, the question of how to exploit this type of data in quantitative methods is generally not trivial. On one hand, textual data may require human interpretation to be used meaningfully and its use in quantitative methods is not straightforward. On the other hand, when evaluating an algorithm, textual data can be useful to provide insight in the results.

In this paper, we first show how it is possible to use textual data in regional geography, and more precisely how to extract textual distances and use them in an adapted clustering algorithm. Secondly, we address the question: how to interpret the clusters obtained from the algorithm in view of, textual and regional characteristics, and using expert knowledge? From a geographical perspective, this second idea follows (Grady and Funka-Lea 2004), which argues that fully automated spatial data analysis does not exploit the advantage of the practitioner’s input performing the classification. Indeed, a person has to evaluate the results of any automated procedure without knowing exactly how the latter was really performed. Even more, the similarity between administrative entities depends on the points of view. For example a territorial network admits several “valid” classifications corresponding on the nature of the analysis, interest or study objectives. Thus, the knowledge provided from the practitioner can be included by specifying in a clustering task, initial memberships to infer the segmentation in a certain aim with keeping the advantage of automated approach. Also, the memberships yielded by the method can be analysed by the practitioner to identify interesting spatio-textual patterns as well as used to refine the initial membership of the algorithm; leading to an iterative clustering approach.

Methodologically this paper uses “Soft Textual Cartography”, as previously developed in (Egloff and Ceré 2018). Textual information is used with the method of regional semi-automated soft clustering proposed by Ceré and Bavaud (Ceré and Bavaud 2017; 2018). That implements the combination of spatial configuration and features distances in an image segmentation framework (see (Youssef Mourchid and Cherifi 2017) for a conceptually comparable approach) to perform semi-automated regional segmentation.

We improve the results as presented in Egloff and Ceré (Egloff and Ceré 2018). Applying the method on a larger dataset composed by all the municipalities of Switzerland. It furthermore, emphasises the role of the initial memberships in the iterative procedure. Also, the analysis of the results is clarified by means of correspondence analysis (CA) between different memberships. For the validation of the obtained memberships we use an official classification provided by the Swiss Federal Statistical Office (FSO).

The paper is structured as follows: section “Data” introduces the basic ingredients necessary for the “soft textual cartography” and the data used for the illustration of the method. Then, in section “Soft Textual Cartography” we introduce the heart of the method explaining: the extraction of the weighted spatial network, the textual distance obtained from topic modelling on the corpus, the spatial autocorrelation and finally, the clustering algorithm itself. In section “Parameter choice and initial conditions”, different initial memberships used to test the model are described, among which the official classification. Section “Results” presents a method to evaluate membership association and analyses some results obtained by the algorithm and compares it to a classical approach. Finally, section “Conclusions” draws some conclusions about the usage of the algorithm.

## Data

Soft textual cartography requires a minimal amount of elements (Egloff and Ceré 2018), namely a dataset of *n* regions with relative weights *f*_*i*_>0, ${\sum \nolimits }_{i}^{n}{f_{i}} = 1$, reflecting their surface, population, or description size. Also each region has to be associated with a text, such as a descriptive document, involving a total variety of *N* words. The final element consists in the spatial configuration, which is defined by the binary adjacency matrix *A*=(*a*_*ij*_) of size *n*×*n* with values 1 if *i* and *j* are distinct and neighbours, and 0 otherwise.

Textual data consists of the Wikipedia pages (Wikipedia 2018; DBpedia 2017) of the *n*=2068 municipalities of Switzerland. To keep a spatial continuum, municipalities of Liechtenstein, as well as foreign enclaves (Campione d’Italia and Büsingen am Hochrein) present in the Swiss territory have been included.

**Fig. 1:**
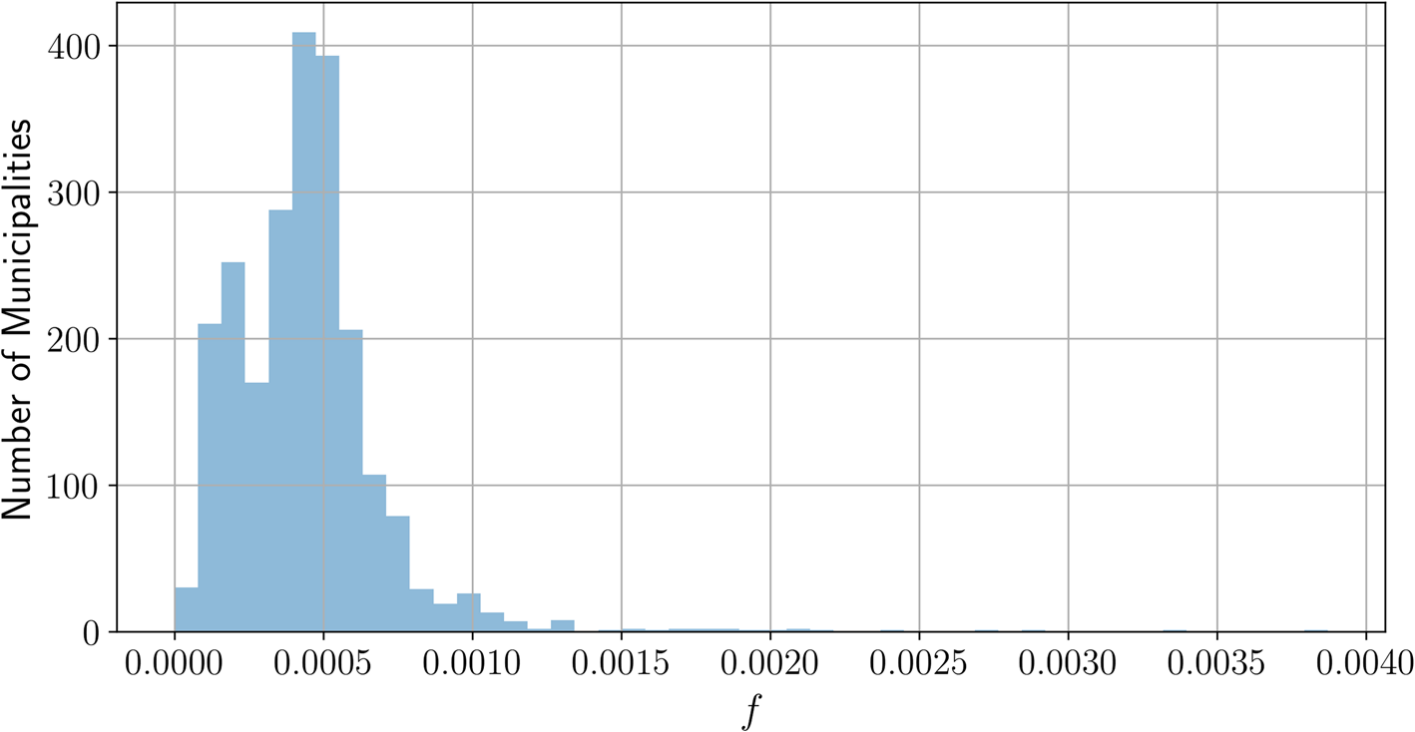
Number of municipalities in function of *f*

Textual sections about important regional personalities as well as external links have been removed. Also, all references to cantons and municipality names have been withdrawn along with the usual stop-words. Finally, low- and high-frequency terms (respectively less than 20 and more than 9000 occurrences) have been also removed (Lu et al. 2017; Xu et al. 2017). Figure 1 shows the resulting weight-frequency *f*. This *f* is reflects the textual volume of information of the Wikipedia pages and defines the relative weight of the municipalities as used in the algorithm.

## Soft Textual Cartography

This section introduces the ingredients involved in the algorithm, in particular the neighbourhood network of the municipalities and the textual distance. Then, with the help of Moran’s *I*, we measure the textual autocorrelation relative to the spatial configuration. Finally, we introduce a particular version of the algorithm leveraging on our previous work (Egloff and Ceré 2018).

### Weighted spatial network

The spatial connectivity between the *n* regions is expressed by a (*n*×*n*) symmetric non-negative *exchange matrix*$ E(A, f, t)=(e_{ij}^{\scriptscriptstyle (t)})$. The latter specifies the joint probability to select the unoriented edge *ij* as prescribed from the time-continuous Markov diffusive process with jump generator *A* at time *t*>0; the so called Laplacian diffusion kernel of machine learning (Smola and Kondor 2003; Fouss et al. 2016) constitutes an unoriented unweighed network. Note that the transition matrix $w_{ij}(t)=e_{ij}^{\scriptscriptstyle (t)}/f_{i}$ is reversible and has a stationary distribution *f*. The weight-compatible $e_{i\bullet }={\sum \nolimits }_{j=1}^{n} e_{ij}=f_{i}$ (Bavaud 2013) diffusive exchange matrix constitutes a weighted generalisation of the unweighed approach using diffusive kernel. Its limit ${\lim }_{\ t\to 0} e_{\scriptscriptstyle ij}^{\scriptscriptstyle (t)}=f_{i}\delta _{ij}$ depicts a network made of disconnected nodes, while ${\lim }_{\ t\to \infty } e_{\scriptscriptstyle ij}^{\scriptscriptstyle (t)}=f_{i}f_{j}$ represents a complete weighted network.

### Textual distance

There are several possible ways to extract distances between the municipalities from textual data. For the approach illustrated a topic distance is defined as follows. First, we define the *N*×*n* term-municipality matrix as the matrix associating each term with its frequency in the document corresponding to each municipality. In a second step we use the Latent Dirichlet Allocation (LDA) (Blei et al. 2003) algorithm to extract the latent *k* topics from the texts, from which the *χ*^2^ distances are finally extracted (see below).

The main idea behind LDA is that a document is conceived as a random mixture over *k* latent topics and each topic a random mixture over the terms or words. The topics obtained from LDA generally are able to regroup words used in similar contexts (semantically correlated or synonyms) into the same topic or theme, namely a set of terms. Consequently, a word possessing more than one sense can belong with a high probability to more than one topic (for example: see “businesses” in topics V2 and V5 in Fig. 3). Furthermore, the theme is mappable to its spatial configuration. The resulting maps can be used for a visual interpretation of geographical socio-economical phenomena. For instance, the topic V4 in Fig. 3 highlights clearly historically established urban regions such as Zurich and Geneva, and is mainly associated to the terms: “city”, “town” and “century”. In this paper we use the Gibbs sampling method to approximate the solution of the LDA to as implemented in the R package topicmodels (Grün and Hornik 2011)).

As the municipalities are in a one-to-one correspondence with the documents: the probability distributions of the municipalities over the topics is defined as the row-normalised (*n*×*k*) document-topic matrix *R*=(*r*_*iq*_), and the probability distributions of the terms over the topics is defined as the row-normalised (*N*×*k*) term-topic matrix *C*=(*c*_*lq*_). The latter permits an interpretation of the topics, whereas the *R* matrix is used to extract topic distances between the regions.

**Fig. 2:**
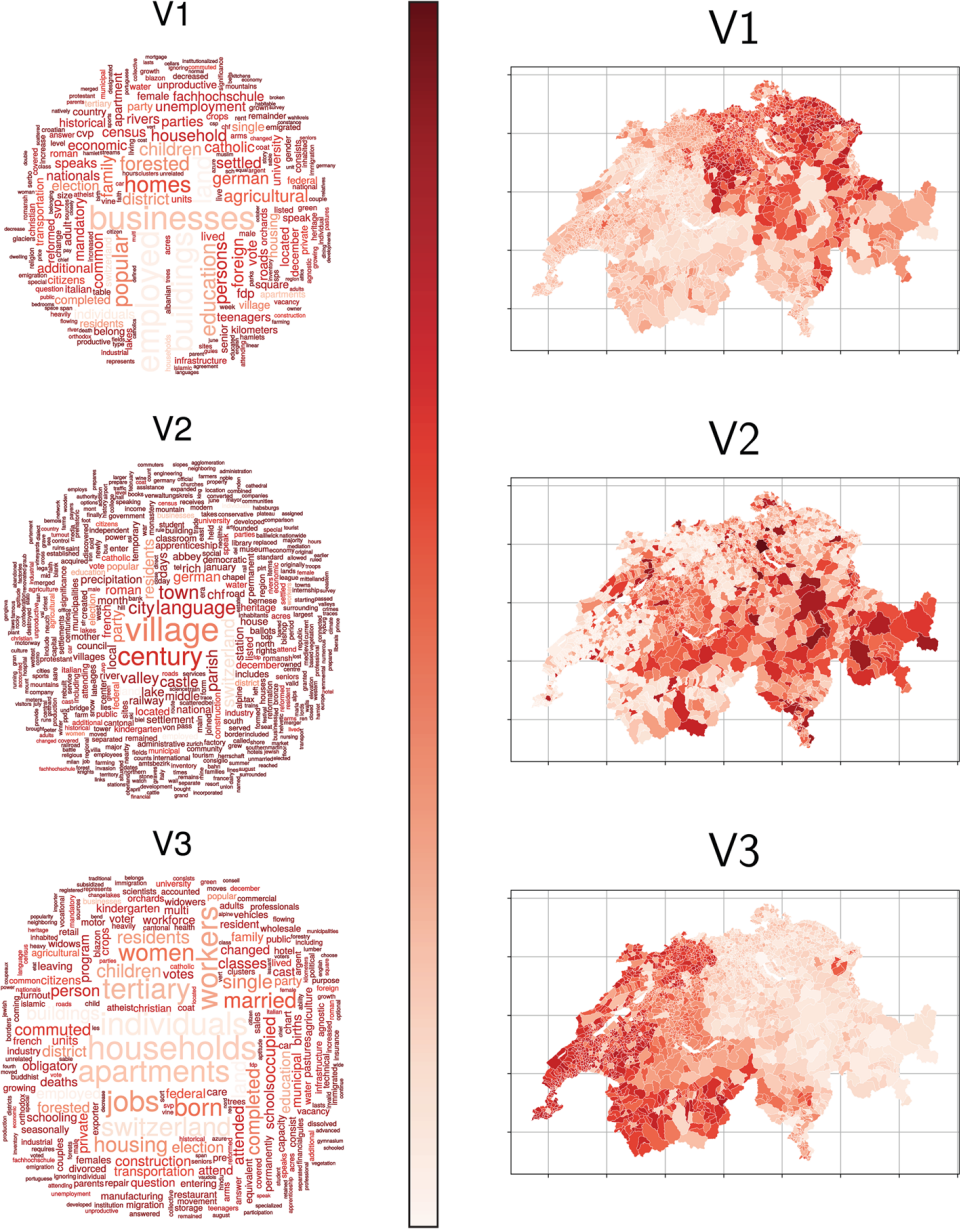
For *k*=3 topics, *Left*: topic wordclouds (Fellows 2014) obtained from the *C* matrix for topic model with parameters: *burning* =4^′^000, 2^′^000 iteration, *thin* =500, *seeds* ={2^′^003,5,63,100^′^001,756}, *nstart* =5 and *best* =true. The colour scale is the inverse of the frequency of the word in the whole dataset (e.g. more specific words are darker than common words) whereas their size represents their importance in the topic. *Right*: related maps with the probabilities of the municipalities over each topic (Map base source: FSO)

**Fig. 3:**
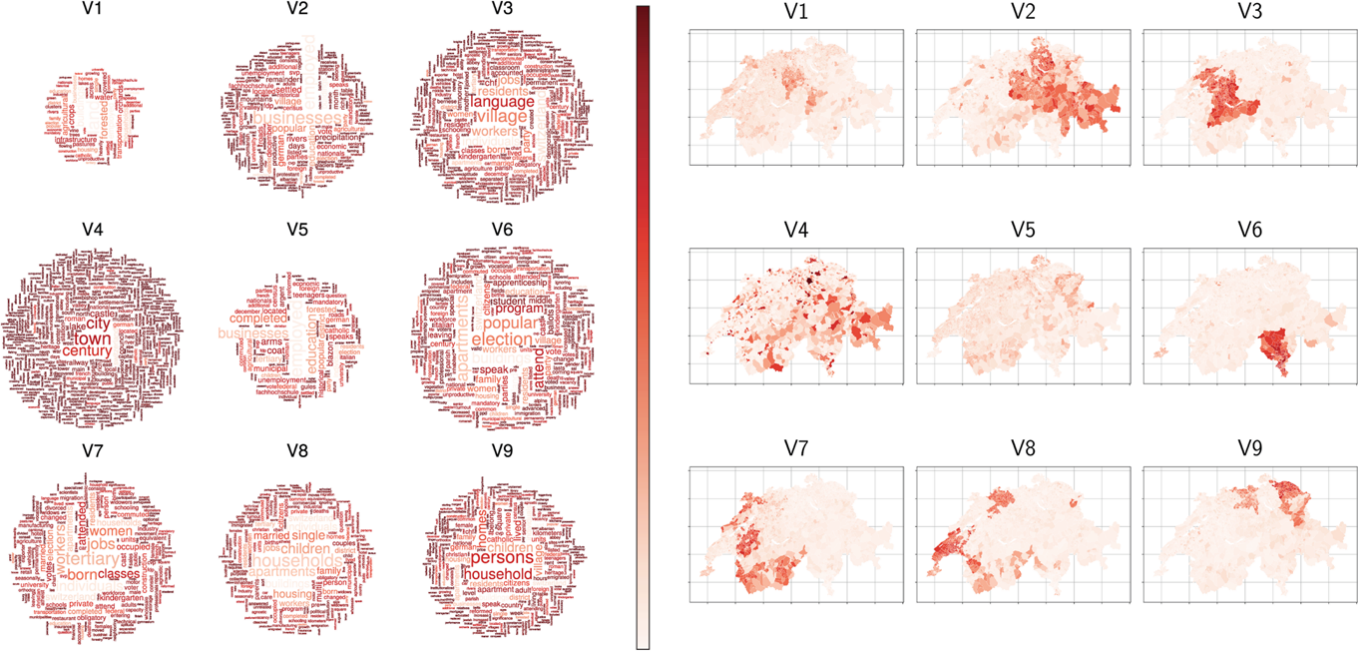
For *k*=9 topics, *Left*: topic wordclouds (Fellows 2014) obtained from the *C* matrix for topic model with parameters: *burning* =4^′^000, 2^′^000 iteration, *thin* =500, *seeds* ={2^′^003,5,63,100^′^001,756}, *nstart* =5 and *best* =true. The colour scale is the inverse of the frequency of the word in the whole dataset (e.g. more specific words are darker than common words) whereas their size represents their importance in the topic. *Right*: related maps with the probabilities of the municipalities over each topic (Map base source: FSO)

To extract the (*n*×*n*) topic-distance *D*=(*d*_*ij*_) from the previously defined municipality-topic matrix *R* the *χ*^2^ distance $d_{ij}^{\chi }={\sum \nolimits }_{q=1}^{k}(r_{iq}-r_{jq})^{2}/R_{k}$ (where $R_{q}={\sum \nolimits }_{i=1}^{n}f_{i}r_{iq}$ are the topic weights) is computed between the topic distributions of the municipalities, i.e. the rows of the *R* matrix. Figures 2 & 3 depict the topic probabilities of the Swiss municipalities; noticeably the topics extracted seem to be spatially autocorrelated.

### Spatial autocorrelation

**Fig. 4:**
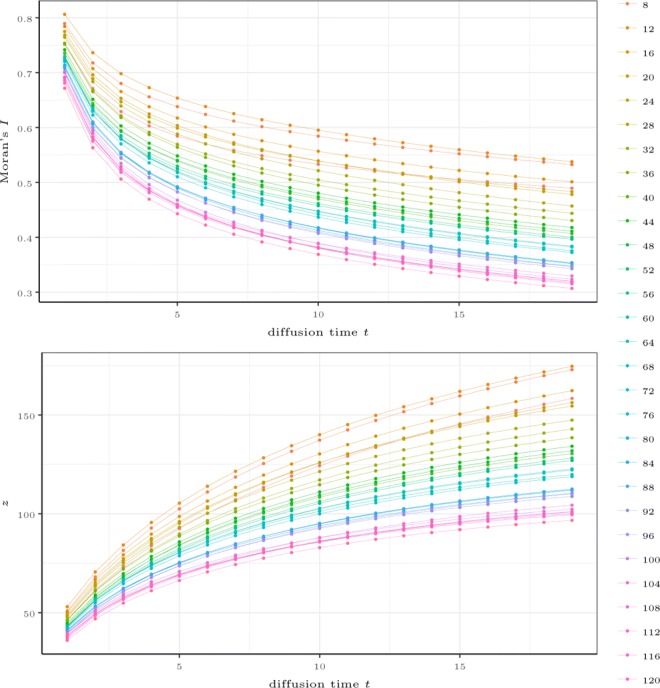
The figures represent *Moran’s I* (*upper*) and *z* (*bottom*) trough the exchange matrix diffusion process at time *t*=1,…,20 using various distances extracted from topic models having *k*=8,12,…,120 topics

Obviously, the basic spatial statistical analysis or classification of an spatial data set makes sense only if a spatial autocorrelation is present. The Moran’*I* provides an index of spatial autocorrelation (Anselin 2010) measuring to which extent the topic-distance *D* is smaller between spatially close municipalities, as defined by the spatial configuration *E*. We use here the weighted, multivariate generalisation of Moran’s *I* where the spatial autocorrelation significance is evaluated with the standardised test value *z* (e.g. (Bavaud 2013; Ceré and Bavaud 2017))

1 $$  I\equiv I(E,D)= \frac{\Delta \ - \ \Delta_{\text{{loc}}}}{\Delta}\qquad \text{with} \qquad z= \frac{|I - E_{0}(I)|} {\sqrt{\text{Var}_{0}(I)}}  $$

2 $$ \text{where} \quad\quad \Delta=\frac{1}{2}\sum\limits_{i,j=1}^{n} f_{i} f_{j} D_{ij} \quad\quad \text{and} \quad\quad \Delta_{\text{{loc}}}=\frac{1}{2}\sum\limits_{i,j=1}^{n} e_{ij} D_{ij}  $$

respectively define the total inertia between all regions and the local inertia between connected regions. The Fig. 4 shows the measured *I*, ranges in [−1,1], where a large positive value is expected when the topic distributions between neighbours are close.

### The Algorithm

As a reminder, the soft clustering method already described in (Egloff and Ceré 2018) is reproduced in this section, with minor adaptations. This approach combines textual information and spatial configuration independently. Notice that the initial membership or partition *Z*^0^ can be used other information (e.g. expert knowledge).

The assignment of *n* objects to *m* groups is represented by the non-negative and row-normalised (*n*×*m*) membership matrix *Z*=(*z*_*ig*_), where *z*_*ig*_ denotes the probability *p*(*g*|*i*) that region *i* belongs to group *g*. In the general soft case, *z*_*ig*_≥0 with ${\sum \nolimits }^{m}_{g=1} z_{ig} = z_{i\bullet } = 1$, whereas *z*_*ig*_=0 or *z*_*ig*_=1 in the hard case.

The soft regional clustering for communities detection (Ceré and Bavaud 2017; 2018) is initialised with initial membership $Z^{0} = \left (z^{0}_{ig}\right)$ and is using expectation maximisation to produce the final assignment. Explicitly, a good membership is defined as local minima of the *generalised discontinuity free energy functional*${\mathcal {F}}[Z]$ from *Z*^0^:

3 $$  {\mathcal{F}}[Z]={\mathcal{K}}[Z]+\beta\Delta_{W}[Z]+\frac{\alpha}{2}{\mathcal{G}}[Z]  $$

where the regularising entropy term ${\mathcal {K}}[Z]$, favouring the advent of soft clustering, is the *mutual information* between the *n* regions and the *m* groups

4 $$ {\mathcal{K}}[Z] = \sum\limits_{ig} f_{i} z_{ig} \ln \frac{z_{ig}}{\rho_{g}} \qquad\qquad \rho_{g}=\sum\limits_{i=1}^{n} f_{i}z_{ig}  $$

where *ρ*_*g*_ is the group weight. The second term $\Delta _{W}[Z]={\sum \nolimits }_{g=1}^{m}\rho _{g}\Delta _{g}$ is the *within-group inertia* relatively to the topic distances, whose presence supports the constitution of group of regions homogeneous enough relatively to the topic distributions, where (Bavaud 2009)

5 $$  \Delta_{g} =\frac12\sum\limits_{ij}f_{i}^{g}f_{j}^{g} D_{ij} \qquad\qquad f_{i}^{g}=p(i|g)=\frac{f_{i}z_{ig}}{\rho_{g}}  $$

The third *discontinuity* term ${\mathcal {G}}[Z]={\sum \nolimits }_{g=1}^{m}\rho _{g}^{-1} \varepsilon [z^{g}]$ and $\varepsilon [z^{g}]=\frac 12{\sum \nolimits }_{ij}e_{ij}(z_{ig}-z_{jg})^{2}$, insures the spatial continuity of the group memberships. As for ${\mathcal {K}}[Z]$, the “spatial energy” ${\mathcal {G}}[Z]$ favours the constitution of soft clusters, in contrast to the “feature energy” *Δ*_*W*_[*Z*] which favours *hard* memberships obeying *z*_*ig*_=0 or *z*_*ig*_=1 (Bavaud 2009).

The parameter *β*>0 controls the influence of topic distances, while *α*=0 coincides with the soft k-means algorithm based on spherical Gaussian mixtures.

Minimising the free energy functional (3) is performed by cancelling the first-order derivative under the conditions *z*_*i*∙_=1 and yields:

6 $$  z_{ig}=\frac{\rho_{g} \exp\left(- \beta D_{i}^{g}+\alpha\rho_{g}^{-1} ({\mathcal{L}}z^{g})_{i}-\frac{\alpha}{2}\rho_{g}^{-2} \varepsilon[z^{g}]\enspace\right)}{{\sum\nolimits}_{h} \rho_{h} \exp\left(- \beta D_{i}^{h}+\alpha\rho_{h}^{-1} ({\mathcal{L}}z^{h})_{i}-\frac{\alpha}{2}\rho_{h}^{-2} \varepsilon[z^{h}]\right)}  $$

where $D_{i}^{g}$ the standardised^1^ squared Euclidean dissimilarity from *i* to the centroid of group *g* and $({\mathcal {L}}z^{g})_{i}$ is the *Laplacian* of membership *z*^*g*^ at region *i*, comparing its value to the average value of its neighbours as defined by the matrix *W* - an ingredient typical of *label propagation models*.

Equation (6) is solved iteratively until convergence. The choice of the initial membership matrix *Z*^0^ is discussed in section “Parameter choice and initial conditions”. The hardness of the final membership matrix *Z*^*∞*^ can possibly be measured by the value of the mutual information ${\mathcal {K}}[Z^{\infty }]$. Also, the point-wise conditional entropy $H(G|i)=-{\sum \nolimits }_{g} z^{\infty }_{ig}\ln z^{\infty }_{ig}$ (where *G* denotes the variable “group”) measures the membership uncertainty of region *i*, and takes on large values for regions located at the group frontiers. Alternatively, the final membership matrix can be further hardened by assigning each region *i* to group $G[i]=\arg \max _{g \in \{1, \dots, m\}} z_{ig}^{\infty }$.

## Parameter choice and initial conditions

To illustrate the algorithm and study the influence of the initial membership *Z*_0_, the following parameter choices were made. First, parameter *k* (the number of topics) was chosen to be the same as the number of groups *m*, thus *k*=*m*. In turn, *m* was chosen to correspond to the numbers of groups presented in the three official municipality classifications issued by the FSO, namely *m*=3, *m*=9 and *m*=25. The value for parameters *β* and *α* of the soft clustering algorithm have been tuned by numerical experimentation. The free parameter *β*, which can be interpreted as the inverse temperature in statistical mechanics, controls the hardness of the classification. The free parameter *α* controls the extent to which the spatial configuration is taken into account. Finally, the parameter *t* controls the age of diffusive process: a low *t* limits the interactions to the nearest neighbours.

**Fig. 5:**
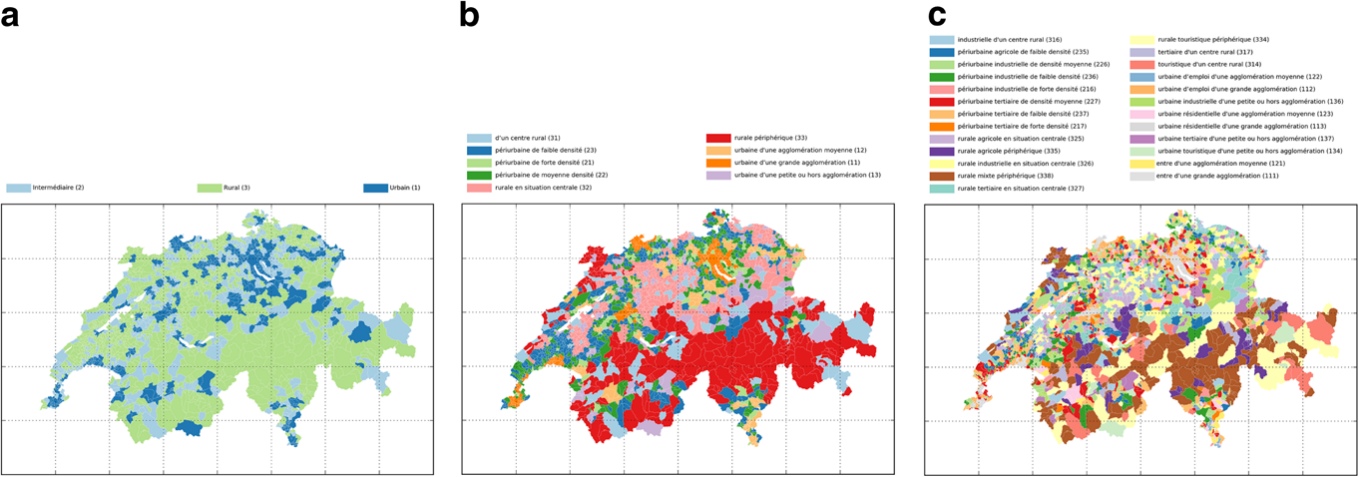
Illustration of the maps of the official classifications, from **a** to **c** the parameters are: *m*=3; *m*=9; *m*=25
- two random memberships (soft and hard) for each municipality *i* to the group *g*, *m*=*k*, where the number of the topics is *m*∈{3,9,25}, see Fig. 6,

To use the clustering algorithm proposed in section “The Algorithm” an initial membership matrix *Z*^0^ is required. To study the impact of the initial membership we went beyond the method proposed in (Egloff and Ceré 2018), where pre-selected municipalities were used based on their atypicality in the correspondence analysis over the topics (their distance towards the mean profile). Hence, three different initial membership attributions:

- three official classifications, *m*∈{3,9,25}, from the FSO based on a urban-rural model, see Fig. 5,

**Fig. 6:**
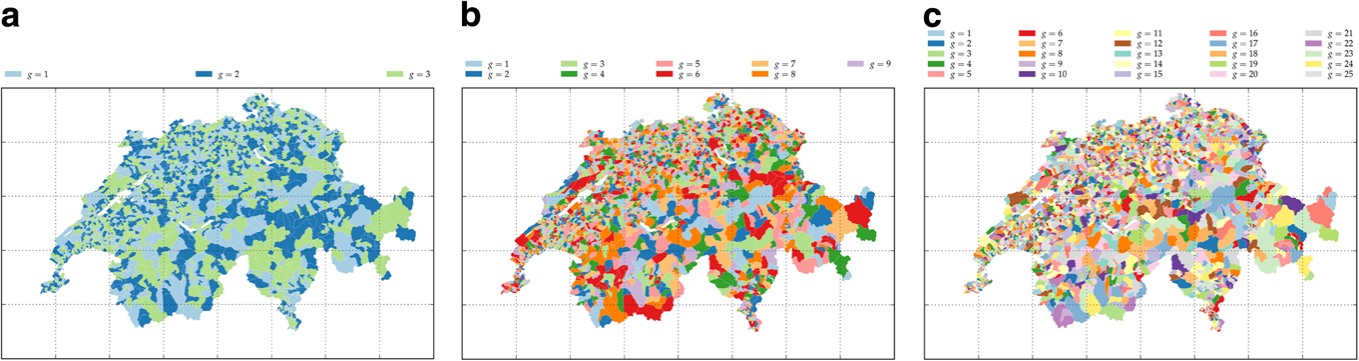
The maps of the groups obtained by hard k-means clustering on the MDS over the generalised *χ*^2^ distance between municipality profiles in the term-municipality matrix. From **a** to **c** the parameters are: *m*=3, *θ*=1.01; *m*=9, *θ*=0.5; *m*=25, *θ*=1.5
- and three hard memberships, *m*∈{3,9,25}, obtained from the k-means algorithm on the generalised *χ*^2^ distance see subsection “APPENDIX: Generalised chi square distance and term-document distance” obtaining from the region-document matrix, represented in Fig. 7.

**Fig. 7:**
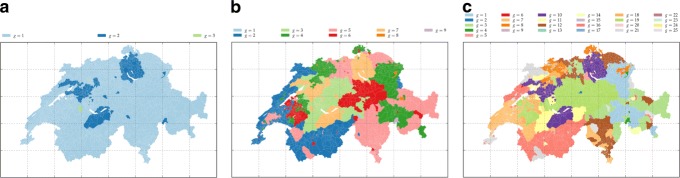
The maps of the groups obtained by hard k-means clustering on the MDS over the generalised *χ*^2^ distance between municipality profiles in the term-municipality matrix. From **a** to **c** the parameters are: *m*=3, *θ*=1.01; *m*=9, *θ*=0.5; *m*=25, *θ*=1.5

### Official classifications

The official municipalities classifications, *m*=3,9,25, of Switzerland (Zecha et al.) (version 2017) is based on the delimitation of the urban space in 2012 based upon morphological (density) and functional (commuting flows) conditions. The *m*=9 categories include the size and the accessibility of the municipalities. The so called rural-urban typology *m*=3 depicts the “Urban (1)”, “Intermediary (2)”, “Rural (3)” municipalities which is based on the classification *m*=9. The *m*=25 categories distinguishes by socio-economic conditions in municipalities. The details of how those typologies have been determined are not further investigated here; those typologies are used here as the “gold standard” to compare the results further obtained.

### Random memberships

For further testing, we first create random memberships where each region is uniformly assigned to groups *g*=1,…,*m*. Three of them are illustrated in Fig. 6.

### Membership based on word-frequency

To test the algorithm further we compute another initial membership based on the term frequencies: we first define a distance based on the term-municipality matrix (defined in 2). To do this, we used the generalised *χ*^2^ distance (see “APPENDIX: Generalised chi square distance and term-document distance”) to compute the distance between the municipalities with respect to their word frequency profile. Figure 7 depicts three examples of groups obtained by submitting the distance obtained to an MDS to which we applied a hard k-means clustering (Hartigan and Wong 1979) with the R package stats (R Core Team 2017). As shown in Fig. 7 this type of clustering has a tendency, depending on the value of *θ*, to create patches of municipalities that either have frequent or rare words in their Wikipedia page. It is not self evident that these patches should be spatially contiguous.

## Results

In this section, we introduce membership association between two memberships, which is later used to compare the results of the algorithm with the official classifications. Then, for each initial membership discussed in section “Parameter choice and initial conditions” we briefly analyse some results. Finally, we compare the present soft textual cartography approach to two classical approaches based on a network obtained from an affinity matrix.

### Membership association

Starting with the initial membership *Z*^0^, the iterative algorithm (6) converges towards a *local minimum* *Z*^*∞*^ of the free energy. *Z*^*∞*^ constitutes a soft membership, which can be further hardened for interpretation purposes, by entirely assigning each municipality *i* to group $G[i]=\arg \max _{g \in \{1, \dots, m\}} (z^{\infty }_{ig})$. On one hand, the iterative process, depending only on the weighted geographical network as well as the the topic-induced distances, should erase in large part the initial attribution *Z*^0^ of municipalities to groups. On the other hand, procedures such as the k-means, soft k-means and their variants are well-known to exhibit sensitive dependence on initial conditions, that is the local minimum *Z*^*∞*^ does in general depend on the initial membership *Z*^0^.

**Fig. 8:**
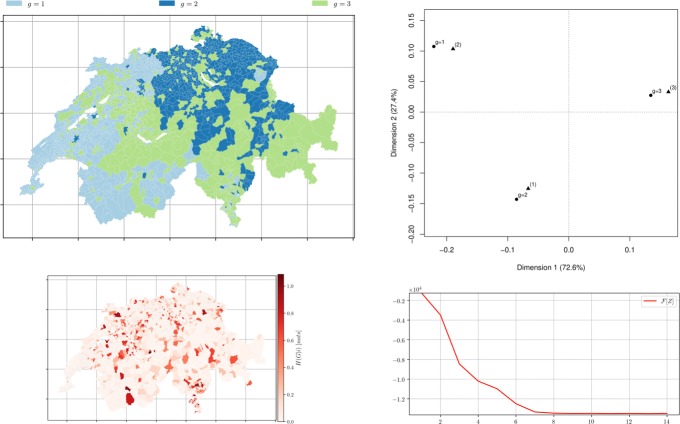
*Municipalities soft clustering on all the topics* depicts the semi-supervised hard assignment obtained from a random membership matrix *Z*^0^ for *m*=3 groups using distance matrix *D* obtained from topic modelling with *k*=3 after 14 iterations. *Left top* Hard membership. *Left bottom* the conditional entropy of topics *H*(*R*|*i*) showing municipality-topic probability distribution uncertainty. *Right top* the CA bi-plot between *Z*^*∞*^ (illustrated by ∙) and the official classification (illustrated by $\blacktriangle $) with *m*=3. *Right bottom* The free-energy plot: decreases as the number of iterative steps increases (*β*=5,*α*=7)

**Fig. 9:**
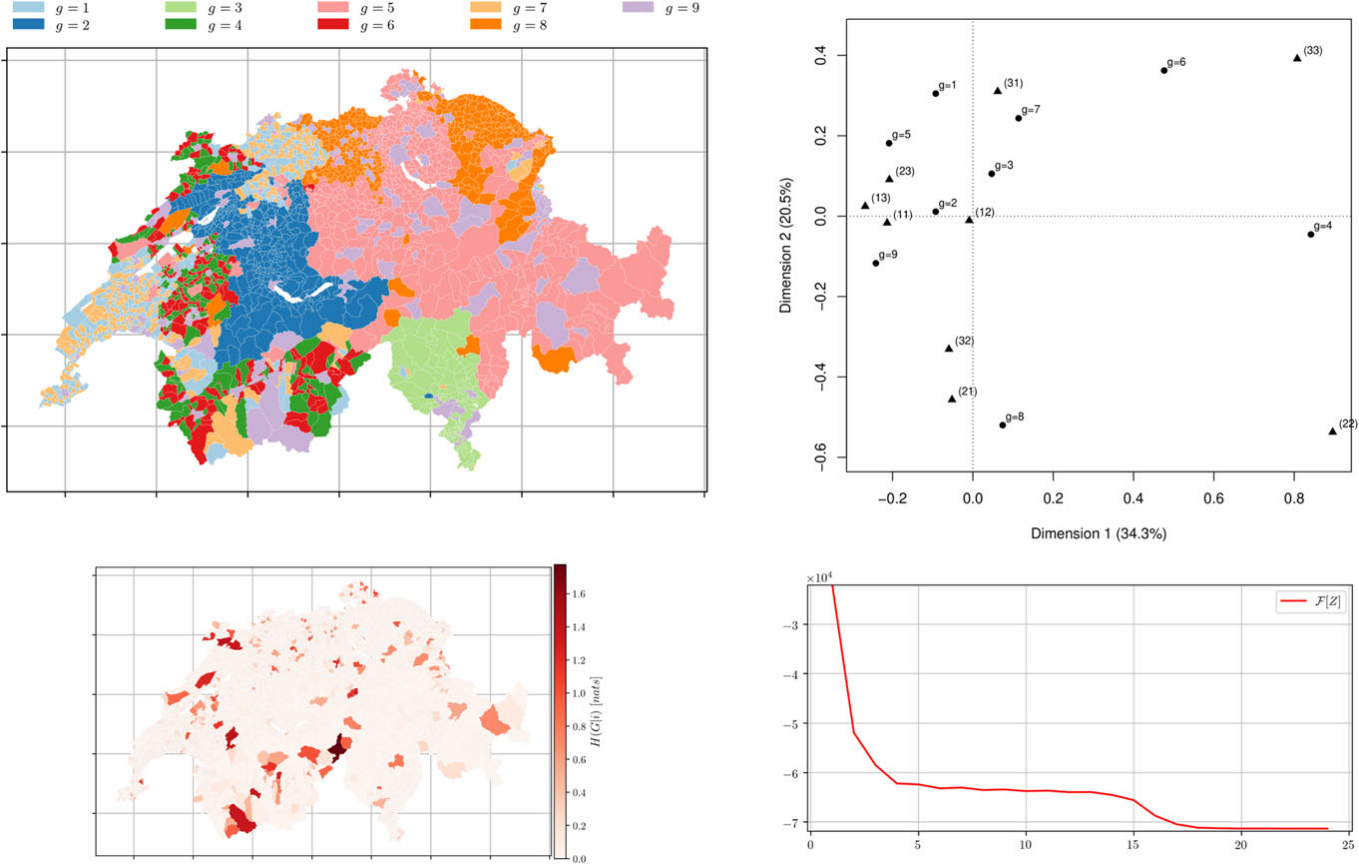
*Municipalities soft clustering on all the topics* depicts the semi-supervised hard assignment obtained from a random membership *Z*^0^ for *m*=9 groups using distance matrix *D* obtained from topic modelling with *k*=9 after 24 iterations. *Left top* Hard membership. *Left bottom* the conditional entropy of topics *H*(*R*|*i*) showing municipality-topic probability distribution uncertainty. *Right top* the CA bi-plot between *Z*^*∞*^ (∙) and the official classification ($\blacktriangle $) with *m*=9. *Right bottom* The free-energy plot (*β*=20,*α*=10)

**Fig. 10:**
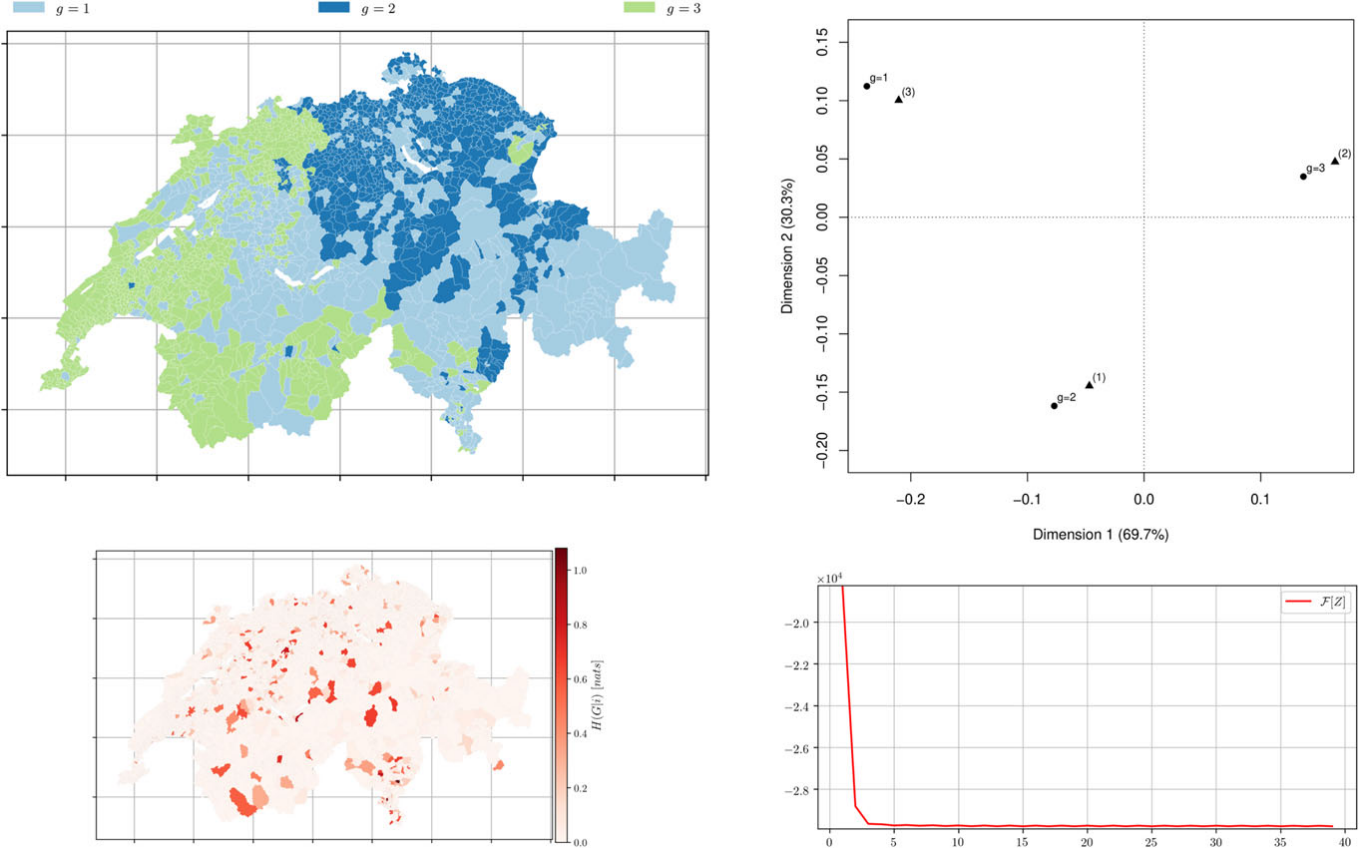
*Municipalities soft clustering on all the topics* depicts the semi-supervised hard assignment obtained from a official classification *Z*^0^ for *m*=3 groups using distance matrix *D* obtained from topic modelling with *k*=3 after 39 iterations. *Left top* Hard membership. *Left bottom* the conditional entropy of topics *H*(*R*|*i*) showing municipality-topic probability distribution uncertainty. *Right top* the correspondence analysis between *Z*^*∞*^ (∙) and the official classification ($\blacktriangle $) with *m*=3. *Right bottom* The free-energy plot (*β*=10,*α*=10)

**Fig. 11:**
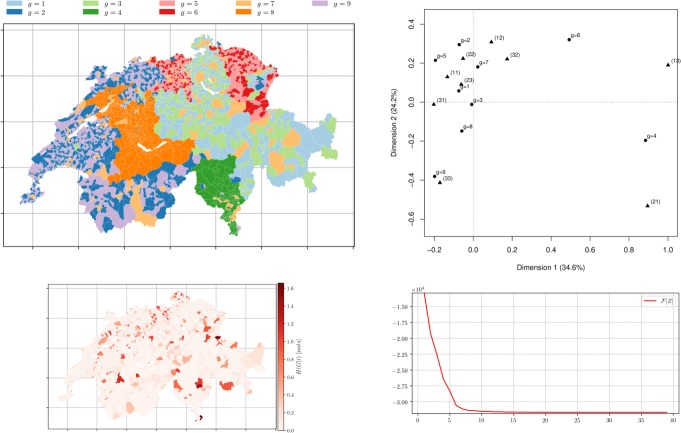
*Municipalities soft clustering on all the topics* depicts the semi-supervised hard assignment obtained from a official classification *Z*^0^ for *m*=9 groups using distance matrix *D* obtained from topic modelling with *k*=9 after 39 iterations. *Left top* Hard membership. *Left bottom* the conditional entropy of topics *H*(*R*|*i*) showing municipality-topic probability distribution uncertainty. *Right top* the CA bi-plot between *Z*^*∞*^ (∙) and the official classification ($\blacktriangle $) with *m*=9. *Right bottom* The free-energy plot (*β*=10,*α*=10)

**Fig. 12:**
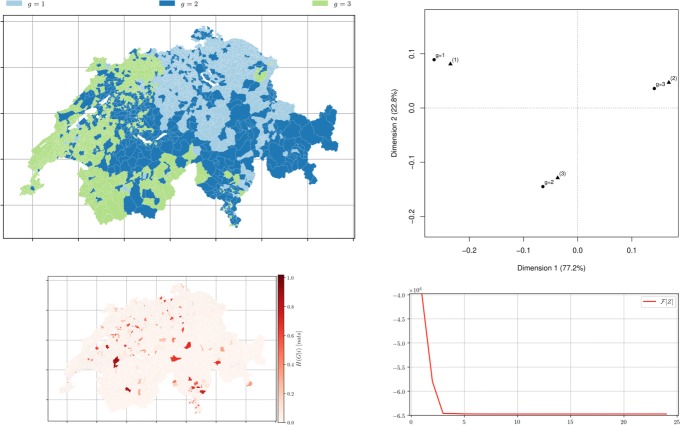
*Municipalities soft clustering on all the topics* depicts the semi-supervised hard assignment obtained from the k-means performed over the therm-frequency distances with *θ*=1.01 on the *Z*^0^ for *m*=3 groups using *χ*^2^ distances *D* obtained from topic modelling with *k*=3 after 39 iterations. *Left top* Hard membership. *Left bottom* the conditional entropy of topics *H*(*R*|*i*) showing municipality-topic probability distribution uncertainty. *Right top* CA bi-plot between *Z*^*∞*^ (∙) and the official classification ($\blacktriangle $) with *m*=3. *Right bottom* The free-energy plot (*β*=20,*α*=10)

**Fig. 13:**
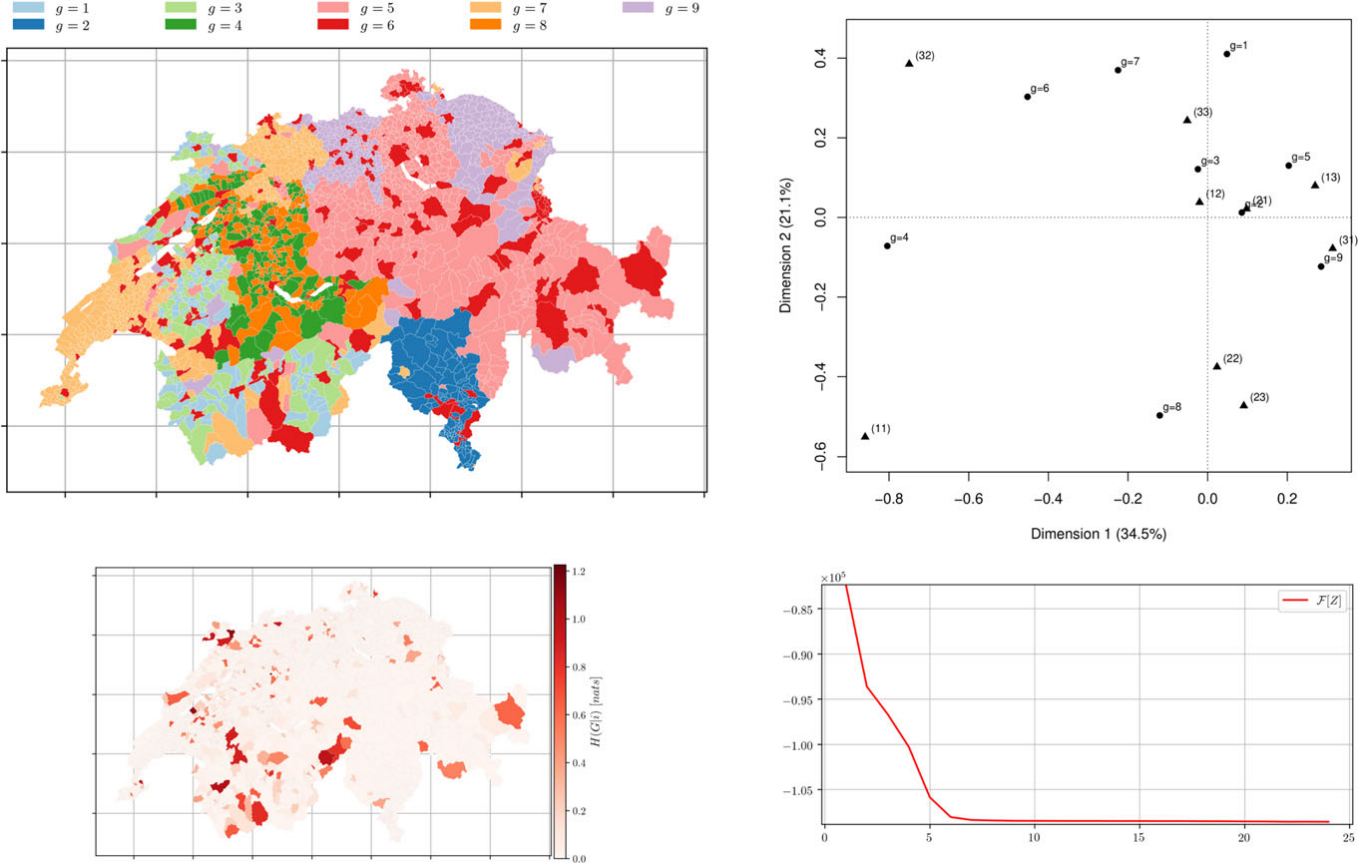
*Municipalities soft clustering on all the topics* depicts the semi-supervised hard assignment obtained from the k-means performed over the therm-frequency distances with *θ*=0.99 on the *Z*^0^ for *m*=9 groups using *χ*^2^ distances *D* obtained from topic modelling with *k*=9 after 24 iterations. *Left top* Hard membership. *Left bottom* the conditional entropy of topics *H*(*R*|*i*) showing municipality-topic probability distribution uncertainty. *Right top* CA bi-plot between *Z*^*∞*^ (∙) and the official classification ($\blacktriangle $) with *m*=9. *Right bottom* The free-energy plot (*β*=30,*α*=10)

**Fig. 14:**
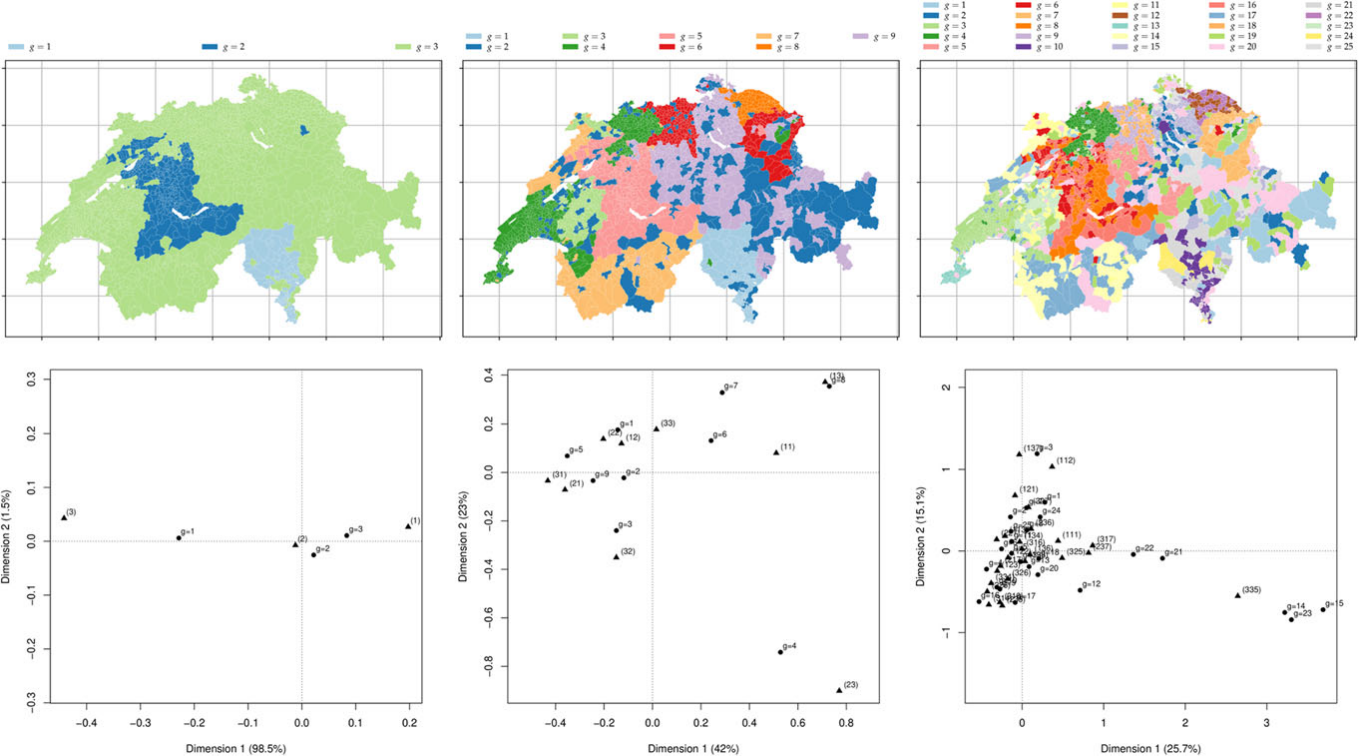
*Top* Municipalities assignments from the spectral clustering with the parameters, respectively left-right, *m*=3,9,25 for 100 iterations applied on the affinity matrix *S* (with: *r*=1.0, *λ*=1.0). *Bottom* the corresponding CA bi-plot

To compare two classifications, *Z* with *m* groups (such as the result of the clustering, hardened or not) and *Y* with $\tilde {m}$ group (such as the official classification), one can first define the $m\times \tilde {m}$*overlap matrix*${\mathcal {T}} = (\tau _{gh})$

7 $$ \tau_{gh} = \sum\limits^{n}_{i=1}f_{i} z_{ig} y_{ih}  $$

whose margins give by construction the group weights *ρ*_*g*_=*τ*_*g*∙_ and $\pi _{h}={\sum \nolimits }_{i}f_{i} y_{ih}=\tau _{\bullet h}$. The matrix ${\mathcal {T}}$ constitutes a normalised version of the contingency table $N {\mathcal {T}}$ (where *N* is the total number of terms in the corpus), whose chi-square attests, expectedly and in all the instances encountered in this work, a very significative dependence between both classifications. Their association can be further investigated by performing a CA on ${\mathcal {T}}$, the resulting bi-plots (Figs. 8, 9, 10, 11, 12, 13, 14) permitting to identify which groups *g*=1,…,*m* of *Z* possibly correspond to which groups $h=1,\ldots,\tilde {m}$ of *Y*, and to which extent.

### Random initial membership

Starting with random memberships as illustrated in subsection “Random memberships” in 2 permits to test how the algorithm behaves when there is not any preliminary information available on the groups.

As depicted in Figs. 8 & 9 the algorithm produced groups which match surprisingly well the official classifications. This result could imply that the different types of municipalities (in the case of *m*=3, the official groups being: “Urban (1)”, “Intermediary (2)”, “Rural (3)”) are reflected by the topics present in the text of their Wikipedia page. For *m*=9 and *m*=25, the match between the official classification and the detected ones is thinner: it could be the case that some types of official groups are less reflected in the topics that the three broad categories of *m*=3, for example “urban of a big agglomeration (11)” and “urban of a mean agglomeration (12)”: those categories make sense from a classification perspective, as they correlate to population and density, but are harder to extract from the Wikipedia description.

### Official groups as initial membership

To test if the algorithm minimises correctly given ideal initial memberships representing the practitioner’s knowledge or an official classification, and to verify the intuition that some official categories are more difficult retrieve from the textual description of the municipalities, the initial membership was set to correspond to the official one. Figure 10, for *m*=3 this initial membership yields, as expected, a better result than the random initial membership. Figure 11, for *m*=9 and *m*=25, the choice of initial memberships is less crucial, and the intuition that some groups proposed by the FSO are harder to recover in the corpus of Wikipedia pages is thus confirmed.

### Initial membership based on word frequency

We explored another approach using memberships obtained by using the hard k-means algorithm on the generalised *χ*^2^ distance (see section “APPENDIX: Generalised chi square distance and term-document distance”) of the municipalities in the term-document matrix. This choice of the initial memberships constitutes an intermediate case between randomness and complete information, and inherits its initial memberships from a distance where the terms can be over-weighted using parameter *θ*. Initial memberships reflect common usage of rare or frequent words (respectively using *θ*<1 or *θ*>1) which can be interpreted as a partial knowledge on the textual similarity between municipalities. The results are consistent with the two cases previously observed (see sections “Random memberships” and “Official groups as initial membership”).

### Comparison with a classical approach

How to combine the spatial configuration *E* of the regions with their textual distances *D* in order to build a *complex* network on which clustering or boundary detection are then applied is not a trivial question.

An alternative, more classical approach is to combine the textual dissimilarity *D*_*ij*_ with the spatial proximity $e_{\scriptscriptstyle ij}^{\scriptscriptstyle (t)}$ used in graph image segmentation (Lézoray and Grady 2012; Solem 2012) which yields the pairwise region affinity *S*=(*s*_*ij*_) as in:

8 $$ s_{ij}=\frac{e_{ij}^{(t)}}{f_{i}f_{j}}\: \exp(-\lambda\: D_{ij})  $$

where the spatial component $e_{ij}^{(t)}/f_{i}f_{j}$ compares the spatial interaction of order *t* between regions *i* and *j* to its expected value under independence. The free parameter *λ*>0 controls the pairwise similarity. The higher *s*_*ij*_, stronger is the interaction along the edge *ij*.

For a general comparison we used the well known community detection algorithm Infomap (Rosvall et al. 2009), from the igraph python package (Csardi and Nepusz 2006) on this network, which turned out to detect *n*/2 communities, irrespectively of the values of parameters. This result could be expected as *S* yields a complete network and the degrees of municipalities are more or less the same.

Another classical community detection algorithm is spectral clustering (von Luxburg 2007). We used the python package scikit-learn (Pedregosa et al. 2011) to perform it on the affinity matrix *S*. Figure 14 shows interesting results, where the correspondence between memberships obtained from spectral clustering and the official classification are already quite good.

## Conclusions

This paper exposes and explores the application of the soft clustering algorithm to the exploration of a spatial and thematic corpus based on the Wikipedia pages of Swiss municipalities. We focused the analysis on the impact of differing initial memberships on the results, in order to explore the robustness of the algorithm; the matching of the latter to the official classifications, permitting to incorporate the practitioner’s knowledge in the analysis, namely the socio-economical and geographical categorisation of municipalities.

This study has permitted, on one hand, to show that the algorithm strongly depends on the textual or topic distances in use, but is otherwise less sensitive to the initial memberships. On the other hand, the association of the groups computed by the algorithm with the official classification of the municipalities is surprisingly high. Finally, the results demonstrate that the Wikipedia pages of the municipalities constitute a corpus that is both spatially and thematically correlated.

This flexible semi-automated approach shows its potential at the exploration stage for large spatio-textual dataset: on one hand the initial membership provides a means to direct the algorithm based on available knowledge, on the other hand this knowledge can be created by interpreting the results. For this task, the interpretation of the topics with respect to their spatial configuration (e.g. geographical) and their defining words is of great value. Consequently, the algorithm can be used as a part of a semi-automatic iterative clustering retaining both aspects of the regions, namely their textual content and their spatial configuration.

## APPENDIX: Generalised chi square distance and term-document distance

The generalised *χ*^2^ distance defined in (11) provides a parameter *θ* which enables to control if the distance should be more sensible to high or low frequencies in the distributions. To define this distance let *U*=(*u*_*il*_) be the (*n*×*N*) document-term matrix, counting the number of occurrences of term *l* in document *i*. The relative document-weights *f*, term-weights *v* and quotients *η* are

9 $$ f_{i}=\frac{u_{i\bullet}}{u_{\bullet\bullet}} \qquad\qquad v_{l}=\frac{u_{\bullet l}}{u_{\bullet\bullet}} \qquad\qquad \eta_{il}=\frac{u_{il}\: u_{\bullet\bullet}}{u_{i\bullet}\: u_{\bullet l}}  $$

The *χ*^2^ distance between documents *i* and *j* is

10 $$ d_{ij}=\sum\limits_{l}v_{l} (\eta_{il}-\eta_{jl})^{2} \text{.}  $$

And the generalised *χ*^2^ distance is defined as:

11 $$  d_{ij}=\sum\limits_{l}v_{l} (\varphi(\eta_{il})-\varphi(\eta_{jl}))^{2} \text{ where} \varphi(\eta) \text{ is any increasing function. }  $$

By construction *d*_*ij*_ defines a squared Euclidean distance between documents *i* and *j*, thus Multidimensional Scaling (MDS) (Bavaud 2004) can be performed.

For instance consider *φ*(*η*)=*η*^*θ*^ with *θ*≥0. The case *θ*=1 yields the usual *χ*^2^ distance. *θ*>1 overweights the contribution of frequent terms, and *θ*<1 overweights the contribution of rare terms. The case *θ*=1/2 yields the so-called *Hellinger distance* (Deza and Deza 2009), and *θ*→0 yields the *presence-absence dissimilarity*:

12 $$ {\lim}_{\theta\to 0+}d^{(\theta)}_{ij}=V_{ij^{c}}+V_{i^{c}j}  $$

where $V_{ij^{c}}={\sum \nolimits }_{l; l\in i, l\notin j}v_{l}\phantom {\dot {i}\!}$ is the total weight of terms present in *i* but not in *j*, and $V_{i^{c}j}\phantom {\dot {i}\!}$ is defined analogously.

## Abbreviations

CA: Correspondence analysis. 2, 10–13
FSO: Swiss federal statistical office. 2, 5, 6, 8, 14
LDA: Latent dirichlet allocation. 4
MDS: Multidimensional scaling. 9, 15

## Footnotes

1 *D* is divided by $\Delta =\frac {1}{2}\sum _{i, j=1}^{n} f_{i}f_{j}D_{ij}$ which amounts to recalibrate the value of the free parameter *β*.
